# Demographic Factors, Cumulative Stressors, and Psychological Well-being

**DOI:** 10.1192/j.eurpsy.2024.1257

**Published:** 2024-08-27

**Authors:** M. Theodoratou, A. Petrou, A. Kalaitzaki

**Affiliations:** ^1^Social Sciences, Hellenic Open University, Patras, Greece; ^2^Health Sciences, Neapolis University Pafos, Pafos, Cyprus; ^3^Social Work, Hellenic Mediterannean University, Heraklio, Greece

## Abstract

**Introduction:**

The COVID-19 pandemic and the Ukrainian war appear to have adverse mental health effects. These global crises have raised concerns about the long-term psychological well-being of individuals across different demographic groups.

**Objectives:**

The objective of this study was to evaluate the cumulative mental health effects of the COVID-19 pandemic and the Ukrainian war, emphasizing the relationship between demographic factors and mental health outcomes.

**Methods:**

This was a cross-sectional online survey using convenience and snowballing methods of recruitment. A sample of 170 participants completed demographic questions and Likert-scale assessments using a range of psychometric scales for measuring general psychological distress, perceived stress, personal resilience, traumatic life events, emotional and social effects of trauma, and potential growth after trauma. Participants were requested to respond to the traumatic experiences of the COVID-19 pandemic and the Ukraine war.

**Results:**

Gender differences were evident, with women reporting higher levels of psychological distress and post-traumatic growth.  Family size had a negative correlation with psychological disturbance. Family status exhibited a positive correlation with traumatic event recall. Specifically, individuals who were either unmarried or divorced demonstrated increased memory recall for such events and levels of psychological distress. Conversely, participants in married or cohabiting relationships displayed diminished recall and lower psychological distress levels.Financial strain strongly correlated with compromised psychological well-being.

**Conclusions:**

These findings highlight the association of demographic factors with cumulative stressors, underscoring the importance of personalized psychosocial interventions. Such interventions can enhance mental well-being and resilience in adversity, ultimately promoting improved psychological health.

**Disclosure of Interest:**

None Declared

